# Awareness and low uptake of post exposure prophylaxis for HIV among clinical medical students in a high endemicity setting

**DOI:** 10.1186/s12889-015-2468-9

**Published:** 2015-11-06

**Authors:** Leopold Ndemnge Aminde, Noah F. Takah, Jean Jacques N. Noubiap, Maxime Tindong, Calypse Ngwasiri, Ahmadou M. Jingi, Andre Pascal Kengne, Anastase Dzudie

**Affiliations:** Clinical Research Education, Networking & Consultancy, Douala, B.P. 3480 Cameroon; Internal Medicine Unit & HIV Treatment Centre, District Hospital Nanga-Eboko, Nanga-Eboko, Cameroon; Global Health Systems Solutions, Limbe, Cameroon; Department of Medicine, Groote Schuur Hospital and University of Cape Town, Cape Town, South Africa; Medical Diagnostic Center, Yaounde, Cameroon; Faculty of Health Sciences, University of Buea, Buea, Cameroon; Faculty of Medicine and Biomedical Sciences, University of Yaounde I, Yaounde, Cameroon; Non-Communicable Disease Research Unit, South African Medical Research Council, Cape Town, South Africa; Department of Medicine, General Hospital Douala, Douala, Cameroon; Department of Medicine, Faculty of Health Sciences, University of Cape Town, Cape Town, South Africa

**Keywords:** Knowledge, Practice, Post exposure prophylaxis (PEP), HIV, Medical students, Cameroon

## Abstract

**Background:**

Adequate knowledge and practices on post exposure prophylaxis (PEP) for HIV among health care providers are crucial for HIV prevention. However there is limited data on PEP knowledge and practice from developing countries where the burden of HIV infection continues to increase. We assessed the knowledge of clinical medical students on PEP, their practices in response to occupational exposure to HIV, as well as the determinants of good knowledge on PEP.

**Methods:**

A cross-sectional study was conducted in November 2014 involving 154 consecutively recruited clinical medical students (4^th^-6^th^ year undergraduates). Data were acquired using a structured questionnaire. Knowledge on PEP was assessed using a questionnaire comprising 25 questions and categorized as: good (20 or more correct answers), moderate (13–19 correct answers) and poor (12 or fewer correct answers).

**Results:**

For the 154 students included (57.8 % being male), the mean age was 23.2 ± 2.4 years, and 89 % had heard about PEP for HIV. The majority of students had moderate (61.7 %) and poor (32.5 %) knowledge on PEP. Overall knowledge score increased with increasing level of studies (*p* < 0.05). Only 10 (6.5 %) had had previous training on PEP, most of whom were senior level students (*p* = 0.01). Fifty-four students (35.1 %) knew the appropriate duration of PEP and this awareness increased with level of studies (*p* = 0.001). Of the 81 (52.6 %) who reported occupational exposure to HIV in the past, only 4 (4.9 %) received PEP.

**Conclusions:**

Overall, knowledge on PEP among clinical medical students in this setting was non-optimal with very low uptake PEP. Intensification of HIV curricula to involve PEP as well as continuous medical education programs and workshops are potential avenues to improve awareness in this vulnerable population.

## Background

The World Health Organization (WHO) has reported that about 90 % of occupational exposures to HIV occur in sub-Saharan Africa (SSA) [1]. HIV infection, one of the main communicable diseases on the sub-Saharan African region has been a global challenge over the last 30 years [2]. In Cameroon, according to the 2013 estimates, the prevalence of HIV among adults aged 15–49 years was 4.3 % while in 2009, Cameroon had the highest HIV prevalence in West and Central Africa at 5.1 % [3]. Within Cameroon, the Southwest Region has one of the highest HIV prevalence in the country [4]. The advent of Antiretroviral therapy (ART) has turned HIV infection into a chronic disease, and as such healthcare practitioners are increasingly expected to provide care to people living with HIV infection (PLWHIV) including for unrelated medical conditions [2]. Therefore, work related risk of HIV acquisition will remain for a long time, a threat for health workers in high endemicity areas for HIV infection.

Post exposure prophylaxis (PEP) refers to the use of short term antiretroviral drugs to reduce the risk of HIV acquisition [5]. Indeed, it may require 3 days from exposure for the virus to be detected in lymph nodes, and up to 5 days in blood [6, 7]. This offers a short window of opportunity during which, HIV acquisition following exposure can be prevented through PEP, which inhibits viral replication and halts the irreversible establishment of the infection [8]. Whilst most studies on the efficacy of PEP are derived from animal models [9], retrospective data from prevention of mother-to-child transmission (PMTCT) studies and occupational exposure support the efficacy in human subjects [5] and also among health care providers in Europe and the U.S. [10]. Health care providers are at risk of occupational HIV acquisition worldwide [11, 12] with 3/1000 injuries resulting in HIV transmission after percutaneous exposure from an HIV infected patient in health settings [13]. Several studies have explored knowledge on PEP for HIV among healthcare providers as a whole [14, 15], nurses [16, 17], medical doctors [18, 19], surgical residents [20], midwifery students [21], medical interns [22], and dental students [23]. Medical students also form an integral part of the health care team and are thus at risk of acquiring HIV or other blood borne pathogens during their hospital placements [24]. In Cameroon, current estimates from the National AIDS control committee in 2014, suggest that out of 528,512 individuals needing ART, only 145,038 (27.4 %) were actually on ART suggesting a very low ART coverage [25]. It is thus important for health care providers including medical students to have adequate knowledge on PEP for HIV to protect themselves prior to starting their life long career, as occupational exposure to HIV has been shown to cause anxiety and stress among healthcare providers and their families [26].

This study was conducted to assess the knowledge and practices, and investigate the determinants of good knowledge on post exposure prophylaxis for HIV among clinical medical students in Cameroon.

## Methods

### Study design and setting

We conducted an observational cross-sectional study in November 2014 at the Faculty of Health Sciences (FHS) of the University of Buea, in the Southwest Region of Cameroon. The FHS is one of the four public medical schools in Cameroon and in addition to training medical doctors (M.D.), nurses (BSc), medical laboratory scientists (BMLS), it also offers a number of post-graduate programs in public health (MPH), fundamental and clinical sciences, amongst others. Overall medical students (excluding other programs) enrolment in the FHS currently stands at about 700 which are somewhat similar to figures from the other state medical schools.

### Study participants and sampling

Respondents had to be in the clinical years of their medical training (years 4, 5 and 6), and willing to provide a written consent. To consider equal chances of participation and not to influence knowledge level, the students were informed of free participation in a census about a week prior via an oral message during lecture hours. On the said day of census, students were then consecutively approached either in their respective lecture halls or at the practicing/ teaching hospitals for inclusion after obtaining their written informed consent. The study was approved by the Ethics Committee of the Regional Delegation of the Ministry of Health (MOH) for the Southwest Region, and conducted in accordance with the declaration of Helsinki.

### Study procedures and data collection

A structured self-administered questionnaire in English language was adapted from published studies [11, 12, 16, 17]. The questionnaire had three sections; socio-demographic characteristics; assessment of knowledge on PEP; circumstances of exposure and practice of PEP for HIV. The validity of the contents of the questionnaire was established through consultation with experts. The questionnaire included 25 questions on knowledge and 12 questions on practices. Questions on knowledge assessed if the participants had ever heard about PEP; the sources of knowledge; if they had ever received training on PEP; what to do in case of exposure, indications, ART and regimens for PEP for HIV (the preferred regimen in Cameroon is the three drugs regimen though the two drugs regimen is also acceptable from current recommendations [1]). Practice questions assessed what participants did in case of exposure, if the sources of exposure and the exposed were screened for HIV with reasons for not doing so where applicable, if they received PEP (for those exposed) and time windows from exposure to starting PEP.

### Scoring of knowledge of participants

Each of the 25 questions on knowledge was equitably scored (one point for each correct answer and zero otherwise). Points were summed across all questions and respondents who scored 20 or more were categorised as having “Good knowledge”, those who scored between 13 and 19 were considered as having “moderate knowledge” while those who scored 12 or less were considered having “poor knowledge”. To investigate the factors associated with good knowledge, the population was again divided into two groups: participants with Poor knowledge (score < 13) and those with Moderate-to-Good knowledge (score ≥ 13).

Regarding students’ practices, there were twelve questions which assessed circumstances of exposure and practice. The practices were simply evaluated based on accurate answers on practices stipulated by guidelines at the time.

### Data analysis

Data analysis used the IBM-SPSS statistical software v.20 for Windows (SPSS Inc., Chicago, IL). We have summarised continuous variables as medians and 25^th^ and 75^th^ percentiles or means and standard deviation (SD), and categorical variables as count and percentages. Group comparisons used chi square test and analysis of the variance (ANOVA), while factors associated with good knowledge were investigated using logistic regressions. A *p*-value < 0.05 was considered statistically significant.

## Results

### Sociodemographic characteristics

We enrolled 154 medical students in their clinical years during the academic year 2013–2014. Their age ranged from 19 to 34 years with a mean of 23.2 (SD 2.3) years, and 57.8 % (*n* = 89) were male. Fifty-five (35.7 %) were 4^th^ year, 63 (40.9 %) 5^th^ year and 36 (23.4 %) 6^th^ year medical students (Table 1).

**Table 1:** Socio-demographic characteristics of the included medical students from the University of Buea, Cameroon, 2014

Variables	N (%) or Median (25^th^-75^th^ percentiles)
Age	22.5 (21.0–25.0)
Sex	
Male	89 (57.8)
Female	65 (42.2)
Religion	
Christian	145 (94.2)
Muslim	9 (5.8)
Academic level	
4^th^ year	55 (35.7)
5^th^ year	63 (40.9)
6^th^ year	36 (23.4)

### Knowledge on post exposure prophylaxis

The majority of participants (89 %, *n* = 137) had heard about PEP for HIV, with the main source of information being ward rounds (73.7 %, *n* = 101), Table 2. Only 10 (6.5 %) reported having received a training on PEP for HIV, with none being a 4th year student and the majority being 6th year students (*p* < 0.05).

**Table 2:** Sources of and knowledge levels on PEP for HIV among clinical medical students according to level of education at the University of Buea, Cameroon, 2014

Variables and responses	4^th^ Year *N* (%)	5^th^ Year *N* (%)	6^th^ Year *N* (%)	Total *N* (%)	*p*-value
Sources of knowledge					
Heard about PEP (*N* = 154)					0.051
Yes	47 (85)	54 (86)	36 (100)	137 (89)	
No	08 (15)	09 (14)	00 (0.0)	17 (11)	
Source of knowledge (*N* = 137)					
Newspaper/journal	02 (4.3)	01 (1.9)	03 (8.3)	6 (4.4)	0.342
Television	00 (0.0)	00 (0.0)	03 (8.3)	03 (2.2)	0.013
Radio	02 (4.3)	00 (0.0)	00 (0.0)	2 (1.5)	0.142
Seminar/workshop	00 (0.0)	02 (3.7)	01 (2.8)	3 (2.2)	0.436
Ward rounds	30 (63.8)	46 (85.2)	26 (72.2)	101 (73.7)	0.045
PEP training	00 (0.0)	04 (7.4)	04 (11.1)	08 (5.8)	0.081
Can’t remember	17 (36.2)	01 (1.9)	00 (0.0)	18 (13.1)	0.001
Ever had training on PEP (*N* = 154)					0.011
Yes	00 (0.0)	04 (6.3)	06 (16.7)	10 (6.5)	
No	52 (100)	59 (93.7)	30 (83.3)	144 (93.5)	
Knowledge of PEP for HIV					
Overall knowledge score	12.7 ± 3.9	13.7 ± 4.1	15.0 ± 3.9	13.7 ± 4.1	0.031
Overall knowledge level					0.456
Good	01 (1.8)	04 (6.4)	04 (11.1)	09 (5.8)	
Moderate	35 (63.7)	38 (60.3)	22 (61.1)	95 (61.7)	
Poor	19 (34.5)	21 (33.3)	10 (27.8)	50 (32.5)	
What proportion of needle prick injuries from HIV infected individuals result in HIV transmission?					0.001
1/100	18 (32.7)	31 (49.3)	11 (30.6)	60 (39.0)	
1/500	04 (7.3)	08 (12.7)	03 (8.3)	15 (9.7)	
3/1000^a^	19 (34.5)	12 (19.0)	17 (47.2)	48 (31.2)	
Do not know	14 (25.5)	12 (19.0)	05 (13.9)	31 (20.1)	
Which of the following are high risk fluids for transmission of HIV? (multiple answers)					
Breast milk^a^	41 (75.9)	56 (94.9)	33 (97.1)	130 (84.4)	0.001
Urine	00 (0.0)	01 (1.7)	01 (1.7)	2 (1.3)	0.496
Peritoneal fluid^a^	09 (16.9)	17 (27.8)	11 (32.4)	37 (24.0)	0.216
Saliva	07 (13.2)	09 (15.3)	06 (17.6)	22 (14.3)	0.851
Pleural fluid^a^	03 (5.7)	13 (21.3)	07 (20.6)	23 (14.9)	0.046
Cerebrospinal fluid^a^	11 (20.8)	17 (27.9)	10 (29.4)	38 (24.7)	0.584
Faeces	00 (0.0)	01 (1.7)	00 (0.0)	1 (0.6)	0.476
Synovial fluid^a^	03 (5.7)	06 (9.8)	11 (32.4)	20 (13.0)	0.001
Indication for initiation of PEP (multiple answers acceptable)					
Needle prick injury^a^	48 (90.6)	55 (94.8)	36 (100.0)	139 (90.3)	0.156
Splashing of blood/bodily fluid on mucosal surfaces^a^	26 (49.1)	28 (48.3)	25 (69.4)	79 (51.3)	0.094
Rape^a^	37 (69.8)	45 (77.6)	32 (88.9)	114 (74.0)	0.106
Infants born HIV positive mothers^a^	32 (60.4)	20 (34.5)	14 (38.9)	66 (42.9)	0.017
First aid measure to institute following needle stick injury					
Promote active bleeding of the wound	20 (36.4)	18 (28.6)	07 (19.4)	45 (29.2)	0.044
Wash thoroughly with soap and water^a^	25 (45.4)	37 (58.7)	29 (80.6)	91 (59.1)	
Do not know	10 (18.2)	08 (12.7)	00 (0.0)	18 (11.7)	
How soon after needle prick should PEP be started?					0.109
Within 1 h^a^	26 (47.3)	21 (33.3)	20 (55.6)	67 (43.5)	
After 72 h	12 (21.8)	10 (15.9)	03 (8.3)	25 (16.2)	
Don’t know	17 (30.9)	32 (50.8)	13 (36.1)	62 (40.3)	
What is the ideal HIV-PEP regimen following needle stick injury?					0.014
One drug regimen	13 (23.6)	15 (23.8)	22 (61.1)	50 (32.5)	
Two drugs regimen	15 (27.4)	19 (30.2)	06 (16.7)	40 (26.0)	
Expanded three drugs regimen^a^	14 (25.4)	20 (31.7)	07 (19.4)	41 (26.6)	
Don’t know	13 (23.6)	09 (14.3)	01 (2.8)	23 (14.9)	
Which of the following drugs are used in PEP (multiple answers acceptable)					
Zidovudine^a^	32 (72.7)	38 (67.8)	19 (63.3)	89 (57.8)	0.689
Glymepiride	01 (2.3)	00 (0.0)	02 (6.7)	03 (1.9)	0.150
Jevirapine	02 (4.5)	07 (12.7)	00 (0.0)	09 (5.8)	0.065
Lamivudine^a^	07 (15.9)	34 (61.8)	12 (40.0)	53 (34.4)	<0.001
Levamisole	00 (0.0)	00 (0.0)	00 (0.0)	25 (16.2)	---
Stavudine^a^	03 (6.8)	09 (16.4)	05 (16.7)	17 (11.0)	0.307
Famotidine	01 (2.3)	00 (0.0)	00 (0.0)	01 (0.6)	0.378
Nevirapine^a^	30 (68.2)	37 (67.3)	18 (60.0)	85 (55.2)	0.736
Duration of PEP with antiretroviral drugs					0.001
For life	00 (0.0)	02 (3.2)	00 (0.0)	2 (1.3)	
2 weeks	00 (0.0)	02 (3.2)	03 (8.3)	5 (3.2)	
4 weeks^a^	11 (20.0)	21 (33.3)	22 (61.1)	54 (35.1)	
24 weeks	04 (7.3)	08 (12.7)	00 (0.0)	12 (7.8)	
28 weeks	08 (14.5)	02 (3.2)	03 (8.3)	13 (8.4)	
Do not know	32 (58.2)	28 (44.4)	08 (22.2)	68 (44.2)	

^a^Correct answers

The knowledge scores on PEP for HIV ranged between 3 and 22, with a median of 14.5 (25^th^–75^th^ percentiles 11.0–17.0). The overall mean knowledge score was 13.7 ± 4.1 and increased with increasing level of studies (*p* = 0.03). The majority of students had moderate knowledge (61.7 %, *n* = 95), while 50 (32.5 %) had poor knowledge and only 9 (5.8 %) had good knowledge on PEP. Regarding some knowledge items, only 31.2 % of students knew the right proportion of needle prick injuries resulting in HIV transmission and, respectively 130 (84.4 %), 37 (24 %), 23 (14.9 %) and 38 (24.7 %) identified breast milk, peritoneal fluid, pleural fluid and cerebrospinal fluid as a risk fluid for HIV transmission. Most of the students (90.3 %, *n* = 139) correctly identified needle prick as an indication of PEP, but only 51.3 % (*n* = 79) reported splashing of blood or bodily fluid on mucosal surfaces as an indication of PEP. Fourth year students identified infants born to HIV mothers as indication for PEP over sixth and fifth years (*p* = 0.017). Forty-five (29.2 %) students considered promoting active bleeding of a wound following needle stick injury, which is inappropriate, as the first aid measure. However, 6^th^ year students were more aware of the appropriate measures to be undertaken after needle stick injury (*p* = 0.04). Only 67 (43.5 %) participants correctly answered that PEP should be started within 1 h after needle prick. With respect to the ideal PEP drug regimen following needle stick injury, only 41 (26.6 %) participants correctly stated the expanded 3 drugs regimen, amongst whom 5^th^ year students were most aware compared to 4^th^ and 6^th^ year students (*p* = 0.014). Overall, 85 (55.2 %) identified Nevirapine, 89 (57.8 %) identified Zidovudine, 53 (34.4 %) identified Lamivudine and 17 (11.0 %) identified Stavudine accurately as drugs that could be used in PEP. Only 54 (35.1 %) students reported the accurate duration of PEP with antiretroviral drugs. Awareness of the accurate duration of PEP increased with increasing academic level (*p* = 0.001).

**Fig. 1:**
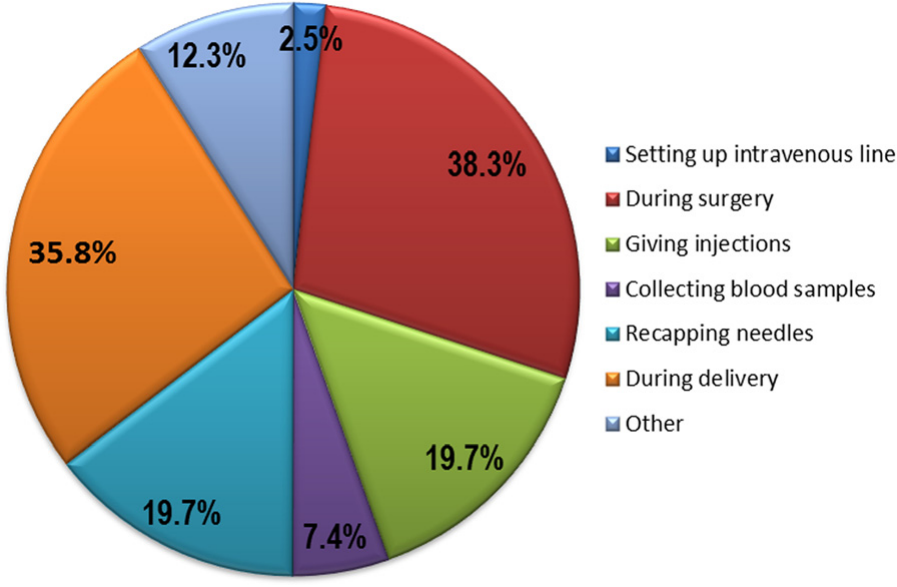
Circumstances of exposure to HIV among clinical medical students at the University of Buea, Cameroon, 2014

Most of the participants (96.1 %, *n* = 148) considered themselves at risk of acquiring HIV and 81 (52.6 %) reported having had occupational exposure to HIV. Needle prick and splashing of blood or bodily fluid on mucosal surfaces were frequently reported (more than 60 %) as the source of exposure. Almost all the students (96.3 %, *n* = 78) who reported a history of occupational exposure to HIV (*n* = 81) had had 1–3 exposures within the last 12 months. The most frequent circumstances of exposure were during surgical procedures (38.3 %, *n* = 31) and delivery (35.8 %, *n* = 29). Of the 81 participants who had been exposed, only 36 (44.4 %) got tested for HIV. Amongst those who did not get tested for HIV (*n* = 45), 22 (48.9 %) assumed the source was HIV negative as their reason for not being tested. There was a very low uptake of PEP (4.9 %, *n* = 4) among the 81 students who reported a history of occupational exposure. In half (*n* = 2) of cases, PEP was received 24 h after exposure. For those exposed participants who did not receive PEP, 13 (16.9 %) declared that they were not aware of the need to receive PEP, 13 (16.9 %) said they were not aware of hospital’s procedures relating to PEP and 13 (16.9 %) did not believe they could be infected by HIV. Only 50% (61.7 %) of the sources of exposures were screened for HIV, 19 of whom were positive (38 %). Table 3 and Fig. 1 demonstrate details of the circumstances of exposure.

**Table 3:** Exposures and practices of clinical medical students regarding PEP for HIV at the University of Buea, Cameroon, 2014

Variables	Frequency (%)
Do you consider yourself to be at risk of HIV acquisition at your work place?	
Yes	148 (96.1)
No	6 (3.9)
Have you ever had occupational exposure to HIV in the past?	
Yes	81 (52.6)
No	73 (47.4)
What type of exposure was it? (*N* = 81)	
Needle prick	31 (38.3)
Splashing of blood/bodily fluid on mucosal surfaces	30 (37.0)
Both Needle prick and splashing of blood on mucosal surface	20 (24.7)
How many exposures have you had in during the last 12 months? (*N* = 81)	
1	40 (49.4)
2–3	38 (46.9)
> 4	3 (3.7)
What were circumstances of exposure? (multiple answers accepted) (*N* = 81)	
Setting up IV line	2 (2.5)
During surgery	31 (38.3)
Giving injections	16 (19.7)
Collecting blood samples	6 (7.4)
Recapping needles	16 (19.7)
During delivery	29 (35.8)
Other	10 (12.3)
If you have had occupational exposure to HIV, did you screen or test for HIV? (*N* = 81)	
Yes	36 (44.4)
No	45 (55.6)
If No, why did you not test for HIV? (*N* = 45)	
Not aware	2 (4.4)
Assumed patient was HIV negative	22 (48.9)
Other reasons	21 (46.7)
Did you receive PEP after exposure? (*N* = 81)	
Received	4 (4.9)
Did not receive	77 (95.1)
What was the time lapse from exposure to which PEP was received after exposure? (*N* = 4)	
< 24 h	2 (50)
> 24 h	2 (50)
Reason for not receiving PEP? (*N* = 77)	
Deemed not necessary	8 (10.4)
ARVs not available	2 (2.6)
Source HIV was negative	24 (31.2)
Not aware of need to take PEP after exposure	13 (16.9)
Not aware of hospital protocol concerning PEP at the time	13 (16.9)
Did not believe I could be infected with HIV	13 (16.9)
Other	4 (5.2)
Post exposure screening of the source of exposure? (*N* = 81)	
Screened	50 (61.7)
Not screened	31 (38.3)
What was the HIV status of the source of exposure? (*N* = 50)	
Positive	19 (38)
Negative	31 (62)

### Determinants of good knowledge for PEP

Previous training on PEP, age, gender, level of medical studies and history of exposure were tested for association with good knowledge on PEP. None of the variables was found to be associated with moderate-to-good knowledge of PEP for HIV in univariable logistic regression models (Table 4).

**Table 4:** Unadjusted correlates of average-to-good knowledge on PEP for HIV among clinical medical students at the University of Buea, Cameroon, 2014

Variables	Odds ratio	95 % CI	*p* value
Age (year)			
< 23	1		
≥ 23	1.35	0.56–2.45	0.476
Sex			
Female	1		
Male	1.17	0.56–2.45	0.671
Religion			
Christian	1		
Muslim	0.51	0.09–2.96	0.450
Academic level			
4th and 5th years	1		
6th year	0.48	0.16–1.43	0.187
Training on PEP			
No	1		
Yes	0.51	0.11–2.39	0.391
History of exposure			
Yes	1		
No	0.75	0.29–1.95	0.553
HIV status of source of exposure			
Positive	1		
Negative	1.74	0.51–5.90	0.373

## Discussion

Our study revealed that the majority of medical students had poor-to-moderate knowledge on PEP. However, adequate knowledge on PEP generally increased with increasing level of studies. Poor practices on PEP for HIV were also observed.

In this study, 89 % of study participants had heard about PEP for HIV. This was lower than reported among health care workers in Gondar, Ethiopia (92.8 %) and in Nigeria (97 %) [11, 12]; although this was higher than the 83.8 % previously reported among nurses in the North West region of Cameroon [17] and the 67.1 % reported among nursing and midwifery students in Hawassa, Ethiopia [21] and the much lower awareness noted among pharmacy students in Malaysia [27]. The main source of knowledge on PEP among medical students in this study was the ward rounds. This is in contrast to findings of Jharn et al. [16] which revealed ‘self-learning’ to be the main source of knowledge.

In most instances, less than a quarter of medical students in this study could identify high risk bodily fluids for HIV transmission. Whilst this was relatively low, it was largely in contrast with other studies among dental students [28] and surgical and anaesthetic residents [29] who incorrectly reported saliva to be a high risk bodily fluid. Over 50 % of students correctly stated that washing site with soap and water was the initial first aid measure to be started following needle prick. This was similar to findings from studies among nurses [16, 17]. Regarding anti-retroviral drugs which could be used for PEP, a few identified Zidovudine which is acceptable especially in regions with limited ARV formulary. However, current recommendations from the updated WHO guidelines of December 2014 on PEP released after the conduct of this study do not suggest Zidovudine again as first line PEP drug [1]. Less than half of the students were aware of the appropriate time for initiating PEP. But the proportion was higher than the 33 % reported among junior doctors [24], 31.6 % among medical interns [22] and far below the 93.7 % reported among family physicians in Nigeria [19]. Significant efficacy of PEP could be obtained if received within 24 h of exposure, albeit can go as long as 48–72 h, after which, it is presumed to have a lower efficacy [5]. Most of these conclusions are however drawn from studies on animal models as evidenced from a recent meta-analysis [30] and not from humans due to ethical implications [5].

About a quarter of medical students knew that the ideal PEP regimen was the three-drug regimen. This is in line with the local adapted country guidelines for HIV PEP in Cameroon [25]. However, the two drugs regimen is likewise efficacious according to recent WHO guidelines [1]. With respect to the duration of PEP, only 35.1 % of medical students knew the duration was 28 days. This was somewhat similar to findings in Nepal [16] though higher than the 15 % recorded by Bairy et al. [31]. Variations in knowledge levels and patterns across these studies could be due to differences in sources of knowledge on PEP, predominantly informal in our study as opposed to formal training in other studies [12], and differences across study populations and settings as well as continued evolution in PEP guidelines during the time-period of conducting these studies.

Regarding students’ practices, almost all students (96.1 %) declared that they were at risk of acquiring HIV in hospital settings, with half admitting to have been exposed in the past. This was in sharp contrast with reports from Ethiopia, Nigeria and Italy [11, 12, 32] though similar to reports from India [33]. The main circumstances of exposure were during surgical procedures, while conducting deliveries, recapping needles and administering injections. Several studies have reported similar circumstances [12, 17, 26,]. In fact, in a study among medical students in Nepal, most exposures/injuries occurred while manipulating needle [34].

Notwithstanding the high exposure rate, only few participants received PEP. This was in line with reports from Nigeria [12], but at variance with reports from Gondar, Ethiopia where three-quarters of exposed participants received PEP, almost all within 24 h of exposure [11]. On the other hand, similar to a study among medical students in Nepal, despite the high rate of exposure to needle prick and sharps, only 11 % received PEP and 19 % did a post exposure serology test [34].

In our study, among reasons for not taking PEP after exposure by students were: “not to be aware of need to take PEP”, “not to be aware of hospital procedures on PEP” and ‘not believing that they could be infected with HIV”. It is possible that stigma, fear, absence of social support, concerns about side effects of ARV as potential barriers for low uptake of PEP for HIV discussed in other studies [35, 36], might in part have accounted for low uptake of PEP in our study. However, most of the reasons in our study were linked to limited knowledge as participants had average-to-poor knowledge on PEP for HIV, which also in part explains the low uptake of PEP in this study. It is noteworthy that studies elsewhere in different populations have suggested that repeated health education and promotion on PEP to improve awareness positively influences uptake of PEP [37]. Secondly, there is no existing university policy or induction program for students on work place safety prior to hospital placements. This in part might account for the non-optimal knowledge and low uptake of PEP. With the growing healthcare demands in Cameroon and increasing numbers of medical schools and/or health training institutions, more students would be faced with this risk and the absence of such policies/programs on work place safety prior to their placements may lead to catastrophic consequences, not only for HIV infection but other and even highly infectious agents like viral hepatitis from exposure to bodily fluids. Currently there exist no published reports on HIV infection rates following occupational exposure in Cameroon and central Africa at large. This would however be interesting to investigate in future studies. Moreover, Pre-exposure prophylaxis (PrEP) for HIV programs have not yet been introduced in Cameroon as opposed to other high income countries [38], which of course together with PEP will constitute a more comprehensive HIV prevention coverage.

The current study is retrospective by nature and therefore carries a high risk of recall bias, which constitutes the main limitation. The study was also conducted in a single medical institution and based on a convenient sample. It may therefore not reflect the full picture of PEP knowledge and uptake among medical students in the country. However, being a pioneer study in the region and owing to the current low ART coverage in Cameroon, the attention our study deserves from health policy makers cannot be overemphasised, with consequent urgent need to improve awareness, training and provision of PEP to curb the growing burden of HIV.

## Conclusions

Our study revealed significant knowledge and practice gaps on post exposure prophylaxis for HIV among clinical medical students in this setting, though generally knowledge level improved with increasing level of studies. Introducing and/or strengthening training modules on workplace safety, organising continuous medical education programs to improve awareness, provision and uptake of PEP for HIV among HCP are needed in this setting. This would allow the development of the health workforce without significantly affecting young people contemplating the medical profession as a career path.

## Abbreviations

HIV: Human immunodeficiency virus
PEP: Post exposure prophylaxis
PLWHIV: People living with HIV
PMTCT: Prevention of mother-to-child transmission
